# Moving Towards Common Data Elements and Core Outcome Measures in Frailty Research

**DOI:** 10.14283/jfa.2019.43

**Published:** 2019-12-12

**Authors:** John Muscedere, J. Afilalo, I. Araujo de Carvalho, M. Cesari, A. Clegg, H.E. Eriksen, K.R. Evans, G. Heckman, J.P. Hirdes, P.M. Kim, B. Laffon, J. Lynn, F. Martin, J.C. Prorok, K. Rockwood, L. Rodrigues Mañas, D. Rolfson, G. Shaw, B. Shea, S. Sinha, O. Theou, P. Tugwell, V. Valdiglesias, B. Vellas, N. Veronese, L.M.K. Wallace, P.R. Williamson, Canadian Frailty Network

**Affiliations:** 1 Kingston General Hospital, Queen's University, Kingston, Canada; 2 McGill University, Montreal, Canada; 3 World Health Organization, Geneva, Switzerland; 4 Fondazione IRCCS Ca' Granda Ospedale Maggiore Policlinico, Università di Milano, Milano, Italy; 5 University of Leeds, Leeds, UK; 6 Healthcare Denmark, Odense, Denmark; 7 Indoc Research, Toronto, Canada; 8 University of Waterloo, Waterloo, Canada; 23 Schlegel-UW Research Institute for Aging, Waterloo, Canada; 9 University of Waterloo, Waterloo, Canada; 10 Canadian Frailty Network, Kingston, Canada; 11 Universidade da Coruña, Coruña, Spain; 12 Altarum, Alexandria, USA; 13 King's College London, London, UK; 14 Dalhousie University, Halifax, Canada; 15 Hospital Universitario de Getafe, Madrid, Spain; 16 University of Alberta, Edmonton, Canada; 17 International Federation on Ageing, Toronto, Canada; 18 Ottawa Hospital Research Institute, Ottawa, Canada; 19 University of Toronto, Toronto, Canada; 20 Centre Hospitalier Universitaire (CHU) de Toulouse, Toulouse, France; 21 National Research Council, Rome, Italy; 22 University of Liverpool, Liverpool, UK

**Keywords:** Frailty, common data elements, core outcome measures

## Abstract

With aging populations around the world, frailty is becoming more prevalent increasing the need for health systems and social systems to deliver optimal evidence based care. However, in spite of the growing number of frailty publications, high-quality evidence for decision making is often lacking. Inadequate descriptions of the populations enrolled including frailty severity and frailty conceptualization, lack of use of validated frailty assessment tools, utilization of different frailty instruments between studies, and variation in reported outcomes impairs the ability to interpret, generalize and implement the research findings. The utilization of common data elements (CDEs) and core outcome measures (COMs) in clinical trials is increasingly being adopted to address such concerns. To catalyze the development and use of CDEs and COMs for future frailty studies, the Canadian Frailty Network (http://www.cfn-nce.ca; CFN), a not-for-profit pan-Canadian nationally-funded research network, convened an international group of experts to examine the issue and plan the path forward. The meeting was structured to allow for an examination of current frailty evidence, ability to learn from other COMs and CDEs initiatives, discussions about specific considerations for frailty COMs and CDEs and finally the identification of the necessary steps for a COMs and CDEs consensus initiative going forward. It was agreed at the onset of the meeting that a statement based on the meeting would be published and herein we report the statement.

## Background

The World Health Organization defines frailty as “a clinically recognizable state in which the ability of older people to cope with everyday or acute stressors is compromised by an increased vulnerability brought by age-associated declines in physiological reserve and function across multiple organ systems.”(1) Improving outcomes, meeting the needs of those living with frailty, and ensuring the best use of limited resources pose challenges for healthcare systems (2). In spite of the increasing number of frailty publications, high-quality evidence for decision making is often lacking because a) participants living with frailty are often excluded from clinical trials; b) studies enrolling older adults rarely consider the differential impact of frailty and c) frailty is often poorly measured or characterized (3, 4, 5).

To better understand the state of frailty research, it is informative to examine frailty randomized controlled trials (RCTs). Although RCTs are regarded as providing the highest quality of evidence, the number of trials primarily enrolling individuals with well-characterised frailty using validated measures is relatively low (6, 7). A recent systematic review of 209 trials conducted to date found that the majority did not use validated frailty criteria and were inconsistent in characterizing frailty, including variable description of function, medication utilization, quality of life, and cognitive status (8). Poor definition of participant populations lowers the quality of an RCT (9). In addition, outcomes reported in RCTs varied, with function-based outcomes measured by a variety of methods being the most common (10). Other outcomes included changes in severity of frailty as assessed by a variety of criteria and laboratory-based outcomes. Further, the importance of many of these outcomes to persons living with frailty is unknown.

Inadequate descriptions of the populations enrolled including frailty severity and frailty conceptualization, lack of use of validated frailty assessment tools, utilization of different frailty instruments between studies, and variation in the outcomes measured impairs the ability to interpret, generalize and implement the research findings. In addition, the lack of study standardization limits the ability to aggregate evidence through data syntheses and meta-analyses. As a result, when deciding on an intervention/treatment or system change, care providers and decision makers are often left with unanswered questions: does evidence generated in non-frail patients have similar risks and benefits in a person with frailty and to what degree of frailty? Were the patients studied similar to ones in my care? As a consequence, treatments are often applied to those with frailty where evidence for effectiveness or harm is lacking, risking inappropriate resource utilization. Conversely, interventions not effective in non-frail patients may actually be effective in those who are frail but may never be utilized due to a lack of evidence.

The lack of standardization in clinical trials is not unique to frailty studies and has been described for other disciplines and diseases (11). In addition, there have been increasing calls on the part of regulatory agencies and funders to standardize the conduct and reporting of clinical trials (12). The utilization of common data elements (CDEs) and core outcome measures (COMs) in clinical trials is increasingly being adopted (e.g., stroke, rheumatoid arthritis, traumatic brain injury) (13, 14, 15, 16).

To catalyze the development and use of CDEs and COMs for future frailty studies, the Canadian Frailty Network (http://www.cfn-nce.ca; CFN), a not-for-profit pan-Canadian nationally-funded research network, convened an international group of experts to examine the issue and plan the path forward on March 4, 2018. For this meeting, we defined CDEs and COMs as follows. CDEs are a minimum standardised set of data elements that would be collected in all frailty studies including population descriptor elements and standardized frailty assessments. COMs are a minimum standardized list of outcomes to be reported in all relevant clinical frailty studies. In addition to the CDEs and COMs, investigators would be expected to collect any other study specific data/information. The objectives of meeting were to:

1. Enable collaborative learning from the expertise of the attendees
2. Learn from existing data and outcome standardization initiatives
3. Identify opportunities and challenges for the development of frailty CDEs and COMs
4. Develop a path forward for the development of frailty CDEs and COMs

Purposeful sampling for meeting invitees was conducted with the criteria that they had published in the field of frailty, were representative of organizations representing or working with ageing populations/older people, or were representatives of organizations that were active in developing CDEs and COMs. It was agreed at the onset of the meeting that a consensus statement based on the meeting would be published and herein we report the statement.

## Learning from other CDEs and COMs initiatives

Three organizations were invited to present at the meeting. One of the oldest initiatives aimed at standardizing outcomes in interventional trials is the Outcome Measures in Rheumatology Trials (OMERACT) (http://www.omeract.org) (13, 17). Since its inception in 1992, the OMERACT core outcome set has been used in over 1,000 peer-reviewed articles, is currently being utilized in 70% of rheumatoid pharmaceutical studies, and is recognized by the European Medicines Agency (EMA), Food and Drug Administration (FDA) and the World Health Organization (WHO) (18). The Core Outcome Measures in Effectiveness Trials (COMET) initiative brings together stakeholders to develop and apply agreed upon standardized sets of outcomes (http://www.comet-initiative.org) (19). The COMET initiative lists over 300 core outcome measure sets with work being done on a further 188 (20). The International Consortium for Health Outcomes Measurement (ICHOM) is a non-profit organization founded in 2012 whose mission is to unlock the potential of value-based health care by defining global standard sets of outcome measures that matter to patients (http://www.ichom.org) (21). Key concepts that emerged from these organizations which should be implemented in the development of frailty CDEs and COMs are:

1. The conceptual framework and necessary domains both by quantitative and qualitative means through a systematic review of the literature and by surveying the views of patients, caregivers and stakeholders must be defined first.
2. Prior to the adoption of any CDEs and COMs, it is necessary to evaluate their measurement properties and feasibility (22). Each instrument/measure should be evaluated through robust processes on the basis of their diagnostic accuracy, discriminative ability, reliability, validity, feasibility and sensitivity to change. Sensitivity to change for outcomes in the frailty literature has been poorly studied and this should be a key area of future investigation. The preference is for previously developed high-quality instruments that have undergone quality assessment; new instruments should not need to be developed. It is important to evaluate if a measure is difficult or resource intensive to use since it may pose a barrier to its widespread adoption.
3. The conceptualization of hierarchies of data to be collected is necessary. This has been referred to as a data onion with the elements necessary in all studies at the center, upon which are layered additional data elements arising from study type and investigator requirement (23, 24).
4. The process of developing CDEs and COMs needs to include stakeholder input including clinicians, patients/caregivers, patient representatives and decision makers (25). It should include the consideration of two components; what should be measured and how should it be measured? Defining appropriate language and outcome measures that are meaningful to patients and their caregivers orients discussions and their contributions should be highly visible and recognized.
5. A transparent process is necessary to choose CDEs and COMs; the scoring process, definition for consensus and selection process need to be set a priori. International consensus is desirable and Delphi surveys are useful to arrive at consensus in a large number of participants (26).
6. For relevance, outcomes should be tailored to the research question (27). This may extend to the instruments chosen. However, the CDEs should remain the same.

## CDEs and COMs considerations specific to frailty

### Defining frailty and frail populations

The frailty research community has made progress in harmonizing schools of thought as to the underlying conceptualisation of frailty (28). The two main conceptual models characterize frailty as a phenotype (29) or as an accumulation of deficits (30) although other models have also been developed, emphasizing function, bio-psychosocial aspects, and dynamic interaction with environment. The definition of frailty as a state of heightened vulnerability is shared among these models. Frailty measurement tools capture these concepts of frailty, from physical phenotype and performance-based measures, through to judgment and questionnaire-based measures that emphasize the stochastic and multidimensional constructs. In addition, the concept of intrinsic capacity, defined as the composite of physical and mental capacities that underlie an individual, has recently emerged (31). Intrinsic capacity is complementary to the construct of frailty, is inherently multidimensional, and is based on the evaluation of assets (residual reserves/capacities) rather than deficits. The development of measurement tools for intrinsic capacity is proceeding (1). Although the evolving conceptualization of frailty has enriched our knowledge of the complexity of aging, the variety of different measurement tools being used have limited the generalizability of the knowledge generated. Going forward, it will be important for the development of frailty CDEs and COMs that these different concepts are captured to the greatest extent possible.

Current common practices for the identification of frailty in studies include subjective assessment based on care setting such as residence in an assisted-living facility or admission to a geriatric inpatient unit. Although setting-based definitions of frailty may appear to be more objective and settings maybe useful for screening or study population enrichment, they should be avoided as descriptors for assessments of frailty since there often exists heterogeneity between settings, regions, or nations and it has been shown that there is a large degree of variability in the severity of frailty in long-term care home residents (32). In addition, frailty is at times equated with advanced age or the presence of comorbidities; these have all been shown to have poor discriminative ability (33, 34). These characterizations have been used to define frailty in studies and these differences impair the generalizability and evaluation of these studies, particularly for international comparative analyses.

### Challenges in engaging older persons living with frailty and their caregivers

The involvement of older persons living with frailty in the development of CDEs and COMs is challenging due to the high prevalence of cognitive impairment, physical disability and declining health over the long period of time that the development of these initiatives requires (35). While the involvement of caregivers may be more feasible, it is important that older people living with frailty be included as much as possible. An additional challenge is the selection bias inherent in convenience/volunteer samples such that highly engaged older people may be more robust (i.e. less frail) or be of a higher socioeconomic status and have a very different outlook on priorities for their care than those who live with severe frailty and thus not representative of the overall frail population. When recruiting older people for development of CDEs and COMs, self-reported poor health could be used as a surrogate for frailty to avoid the lack of participants who may not have already been identified as frail in their clinical environments (36).

## Path Forward

### Scope of the Initiative

Although it would be desirable to have harmonised CDEs and COMs for both frailty research and administrative data collection, to increase the feasibility and likelihood of success, initial efforts should focus on frailty research of any study design including trials of frailty interventions and purely observational studies, with the aim of harmonizing with administrative data sets in the future. Given the heterogeneity of possible interventions from patient level to system-wide interventions, overarching COMs will likely not be feasible. COMs will need to be tailored to the type of frailty study, interventions or care settings. As an example, in early phase clinical trials, non-patient centered outcomes such as a change in a biomarker may be important, but not as important for later phase trials. In contrast, there was agreement that it will be possible to develop overarching CDEs for frailty studies.

### Feasibility

Overall, there was agreement that this initiative was feasible going forward both in the ability to arrive at CDEs, tailored COMs and the ability to implement them in future research. Implementation will depend on data collection burden and the tools or instruments adopted. Funders of research (e.g. CFN) and health systems funders could also move the process forward by mandating the collection of minimum data sets in funded studies. Although this initiative originated from Canada, it should have worldwide representation since the international community would benefit from improving frailty research and international input would increase its adoption worldwide.

### Tailoring of CDEs and COMs to type of interventional frailty studies

When considering the adoption of CDEs and COMs to studies of frailty interventions, four types can be conceptualized.

1. Type 1. Studies of interventions which aim to modify the trajectory of frailty, i.e. an intervention is applied to those who are fit or identified as being at risk for, or living with frailty and then it is determined whether the intervention is able to modify the trajectory of frailty.
2. Type 2. Studies that do not aim to impact frailty severity but aim to modify the impact of an intervention on a frail population e.g. an intervention is applied to persons identified as being frail and the trial aims to determine if a related outcome (e.g. health-related quality of life, activities of daily living, falls) can be improved.
3. Type 3. Studies of disease-specific interventions conducted where frailty could be an important modifier of the burden or outcome of the intervention, i.e. an intervention is applied to a population of well-characterised frail and non-frail persons, with an a-priori planned analysis of the impact of frailty status on the effects of the intervention.
4. Type 4. Studies of health system-level interventions to re-organize care or practices to improve outcomes in those who are frail, i.e. an intervention is applied to a health system and resultant frailty outcomes determined. The intervention is not the clinical intervention per se but rather the way, by whom, where, or how it is delivered (37).

Although CDEs should be similar between the four types of studies, there was agreement that there would not be primary outcome measures that would apply to all. Nevertheless, standardization of how outcomes were measured and then uniformly applying them across all applicable study types will be important. There are likely some secondary outcomes that would be applicable to all trials such as the severity of frailty and quality of life. For the CDEs, measurement of frailty and population descriptors such as co-morbidities, medication utilization, caregiver burden and physical and cognitive function would be common to all types of frailty trials. Although these are important as population descriptors, many of them when measured post intervention could also serve as outcomes.

### Core outcome measures

COMs adopted in frailty trials need to have been evaluated for their validity, reliability, utility and responsiveness (38, 39). Of particular importance was responsiveness to the intervention or the ability of an outcome to change with the intervention being studied.

There was agreement that death should be a secondary outcome in frailty trials. Although it is easier to measure than other adverse health outcomes, what matters to many older adults is not necessarily living longer, but living better with functional abilities that enable them to maintain a high quality of life (40). The use of death as an outcome stems from a disease-based treatment approach and a transformative approach relevant to the care of older adults living with frailty would be to use a holistic approach based on function. Timing of mortality occurrence is important since it may be modifiable with an appropriate intervention; however, this should be accompanied by measurements of other health characteristics such as quality of life since increased longevity may be associated with a low quality of life (41).

Physical performance measures that are responsive to change (e.g. the Short Physical Performance Battery (SPPB)), and are correlated with quality of life would be an option (42, 43). Some disadvantages are the clinical burden of performance-based tests, possible ceiling effects in healthy older adults, and floor effects among the severely frail and its responsiveness has been called into question (44, 45).

Accompanying clinical outcomes with robust cost-effectiveness analyses, including health related quality of life allowing derivation of health utility estimates, is especially important to influence policy change.

### Common data elements

**Figure 1:**
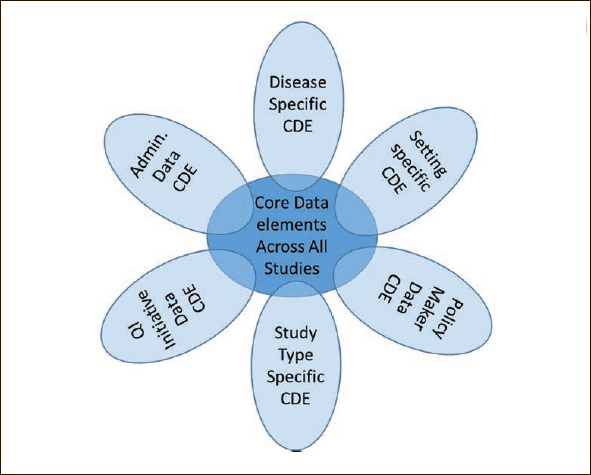
Frailty common data elements flower

CDEs should be collected across all frailty clinical studies. These would form the minimum data set upon which all other data could be collected depending on the type of study, intervention, setting, intent of the study and needs of the investigator (Figure 1) (46, 47). The elements of the core data set would be based upon the domains common to different frailty constructs and possibly intrinsic capacity and would be as minimal as possible to give investigators and stakeholders maximum flexibility.

There was consensus that chronological age-based or age-defined inclusion criteria for studies should be avoided since aging is very heterogeneous and frailty can start earlier in the life course. Conversely, although frailty increases with age, the two are not synonymous with only approximately half of older adults over the age of 85 living with frailty (48). Instead of age, measurement of frailty needs to be understood as an important quantifier of risk of potential adverse outcomes. However, age may be an important determinant of people's values and preferences, and this should be considered when interpreting results.

There was strong agreement that the degree or severity of frailty needs to be reported in all studies whose intent is to study the impact of an intervention. If conducting a study on older patients, there was consensus that the differential impact on the sub-population that is frail be reported since the severity of frailty may determine responsiveness to the intervention. When reporting on frailty, multicomponent frailty measures would be considered best to capture all the aspects/dimensions of frailty.

There was agreement that important domains of CDEs are functional assessment and quality of life. Function is important because it is an expression of multiple intrinsic and extrinsic dimensions which are often considered when assessing frailty. There was also agreement that it is important to shift from a disease-based approach to one rooted in function. Frailty reflects the predisposition to adverse health outcomes that is usually manifested by functional impairment/poor function under stress. There was also agreement that quality of life be measured in all frailty studies.

In addition to CDEs, studies need to be explicit about eligibility criteria and ensure that these are as wide as possible since they are directly related to the generalizability and effectiveness of trials in the real world. Overall, it is essential that CDEs are able to characterize the population and level of frailty and ensure that there is the ability to objectively identify the population in whom it is to be used.

### Harmonization between frailty constructs and intrinsic capacity

**Figure 2:**
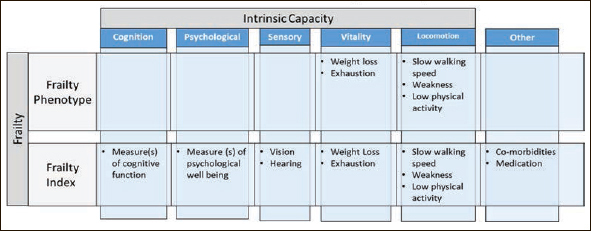
Intrinsic capacity, frailty phenotype and frailty index

There was agreement that harmonizing the frailty constructs and the concept of intrinsic capacity using a domains perspective would be feasible as the underlying domains are similar (Figure 2). This initiative may promote further consensus building on a working definition that most parties can adopt. There was agreement that it would be best to be as inclusive as possible and choosing one model would risk excluding a large group of researchers and health systems. In addition, if the right data elements are collected, a study could be interpreted from a phenotype, a cumulative deficit or multidimensional perspective as in intrinsic capacity. In addition, there was agreement that a domain be included to determine or measure extrinsic characteristics such as social vulnerability. If CDEs could be developed with these principles in mind, it would be an ideal way to move trials forward along with our understanding of frailty and intrinsic capacity.

Frailty can be considered a reflection of diminished intrinsic capacity along the life course. When a high level of functional reserve is present, frailty does not manifest and there exists a high level of intrinsic capacity. When there is little functional reserve, as with disability or high levels of frailty, intrinsic capacity is low. The concepts of intrinsic capacity and frailty are not mutually exclusive and in fact are complementary with many of the domains used to measure intrinsic capacity also used to measure frailty, based on the frailty index approach (49). Frailty in the developed world is associated with advanced age and intrinsic capacity attempts to capture the decline of health trajectories earlier in the life course by using a healthy ageing perspective. However, operationalizing intrinsic capacity in a clinical setting has proven to be challenging and a collaborative, multidisciplinary and integrated approach is needed. This can benefit from the available evidence on frailty where commonalities with intrinsic capacity can be used.

We propose to develop CDEs that allow for the measurement of frailty and are compatible with the intrinsic capacity framework. To combine the chosen measurements for gradation of frailty severity and assessment of longitudinal change, we propose a count-based frailty index. At least 30 metrics would be captured, which is the minimum required for stability of frailty severity, with a representative sampling of the intrinsic capacity domains (i.e. locomotion, vitality, sensory, psychological, cognition) as long as criteria for deficit selection was maintained (50). Using the intrinsic capacity domains to develop CDEs will allow for the ability to develop an optimized list of deficit categories and importantly, would include function and cognition which will likely be important to stakeholders. In addition, the careful selection of deficit categories would allow for multi-dimensional assessment of frailty. These would in turn form the minimum dataset to characterize the populations enrolled in studies. Although the inclusion of 30 items in the frailty index may raise concerns about data collection burden, many studies already collect this amount of data.

The five items of the Fried Phenotype would be similarly captured with CDEs (29). Moreover there is preliminary work to suggest that questions about weakness correlate well with handgrip strength, and thus may obviate the need for such measurements (10). Once the elements in the frailty index are selected, validation of the index would be important including the ability to simultaneously measure frailty through a deficit model, phenotypic model and ability to determine intrinsic capacity. Social parameters are important background information, but these are not usually captured. How this is handled in the future will need to be decided but its incorporation in CDEs is likely important and its characterization could be explored in large datasets (51).

### Patient/Caregiver engagement

Although the challenges of patient's engagement are recognized, patients and caregivers need to be consulted as the process of developing CDEs and COMs proceeds. Using patient or caregiver networks may aid in increasing patient/caregiver participation. Examples of organizations that maybe helpful in this process that were mentioned are: Help Age International (http://www.helpage.org/), an organization which reaches many countries and has a particularly good representation amongst developing nations, the Strategy on Patient-Oriented Research (SPOR) units in Canada (http://www.cihr-irsc.gc.ca), the Picker Institute Europe at Oxford (http://www.picker.org) and the Patient-Centered Outcomes Research Institute (PCORI) (http://www.PCORI.org/engagement) in the United States. In addition there is an increasing need for patient involvement in frailty research and this initiative would be an important springboard for this (52, 53).

## Aspects of CDEs or COMs requiring further input from the broader community

### Scope and Spread of initiative

When and how do we spread the CDEs and COMs initiative? Starting with interventional studies may be most feasible but observational studies are of great value to study populations with frailty and thus their inclusion should be a high priority. If possible and contingent on broader consultation, making this initiative applicable to all frailty research would be highly desirable. Harmonization with administrative data collection should be considered in the future recognizing that this type of data is usually different than what is reported in research studies. However, research ready administrative data will become more available to investigators in the near future making harmonization increasingly important and feasible. Two examples are as follows. The IMPACT statute in the United States will require standardized assessment (e.g. ADLs, IADLs, cognitive function) by the end of 2019 in every person who gets Medicare-funded after-hospital care (https://www.cms.gov/Medicare). In Canada, standardized frailty assessments will be required on all inpatient wards in the near future (https://www.cihi.ca).

### Final selection of domains and Instruments within domains

The final selection of CDE and COM domains and the selection of tools and instruments within the domains will require broad input from the international community and was beyond the scope of this initial meeting. This will be done through a Delphi process that would go out to as many nations as possible with the possibility that respondents could add additional item(s). In this manner geographical differences would be captured. The extent of input will directly influence the degree to which this initiative is broadly adopted.

Collaboration with longitudinal studies on aging across the world will be important since they are using, have developed, or can help validate instruments within domains. These include the Canadian Longitudinal Study on Aging (http://www.clsa-elcv.ca/), the Health and Retirement Study (hrsparticipants.isr.umich.edu/), Survey of Health, Ageing and Retirement in Europe (SHARE) (http://www.share-project.org/), The Irish Longitudinal Study on Aging (TILDA) (tilda.tcd.ie/), Community Ageing Research 75+ (CARE75+) (http://clahrc-yh.nihr.ac.uk/our-themes/primary-care-based-management-of-frailty-in-older-people/projects/the-yorkshire-humber-community-ageing-research-care-study), and the English Longitudinal Study of Ageing (http://www.elsa-project.ac.uk/). These organizations also have significant engagement processes and infrastructure that may help with our patient engagement needs. Also, the World Health Organization has conducted international work on the five domains established in the intrinsic capacity initiative and would be a good resource for this initiative going forward, especially in facilitating consensus building.

### How we do ensure that patient/caregiver voices are heard

While patients and their family/friend caregivers will be in the minority in committees due to practical limitations, we must ensure that their voices are heard and reflected in the final consensus. For example, in the OMERACT initiative, fatigue was identified by patients as an important priority but it did not make the final list of “core outcomes” due to criteria requiring a majority vote for adoption (54). How a patient's priorities are weighted such that they have a higher chance of inclusion in the final measures will require careful consideration and consensus. Paid and unpaid caregiver metrics should be included and be part of the data sets. These metrics will need to be defined and agreed upon.

### Consensus building

Since it will be important to develop consensus across a wide variety of stakeholder groups including clinicians, patients/caregivers, and decision/policy makers, methods for arriving at group consensus will need to be articulated and agreed upon, a priori. These stakeholders need to be from a wide variety of health systems, regions and countries. There are different conceptualizations of frailty and reaching consensus and agreement between the various proponents will be important to ensure widespread adoption.

### When and how should this initiative be harmonized with non-research initiatives?

Although there was broad consensus that this should start as an initiative focused on research, how and when it is harmonized with non-research initiatives such as ICHOM and administrative databases will require further input and discussion. However, if CDEs and COMs are adopted widely for frailty studies, that could influence what is collected routinely. Importantly, harmonizing with routinely collected data, including data definitions, will be important to avoid redundancy.

### Measurement of the environment in which an intervention occurs

Measurement and description of the environment is important since context often has important impact on outcomes. The provider-patient relationships, behaviours, and treatments can all influence the outcome. For example, to interpret a study reporting on frailty outcomes, it would be important to know about the availability of social and community supports. The development of environmental CDEs will require further input from the community.

### Consultation with regulatory authorities

Any measures developed could be useful for registration trials and thus early consultation with regulatory bodies should be part of the process going forward. The EMA recently released a discussion paper describing how to characterize baseline frailty status of older patients enrolled in clinical trials, other than by age (55). The aim of this statement is to ensure that clinical trial populations are representative of the medication users as the benefit-risk balance in older patients may depend on their frailty status. However, the EMA recommended the SPPB and if not possible gait speed, which although are correlated with frailty, are one dimensional (i.e. physical function) measures. To the present, regulatory agencies such as the EMA and FDA have preferred to deal with one dimensional constructs rather than heterogeneous or multidimensional conditions such as frailty. Increasing adoption of frailty CDEs and COMs may encourage regulatory agencies to adopt such measures in regulatory trials leading to better evaluation of efficacy and safety profiles of medications/devices likely to be used in patients living with frailty. Along with this, there should be an increased emphasis on reporting the differential impact of interventions in trials stratified on the basis of frailty status and severity along with increased inclusion of patients living with frailty in registry trials.

### Harmonization with interRAI

Once the domains of frailty CDEs and COMs are agreed upon, if possible, it would be advantageous to harmonize domain instruments with ones commonly collected already. There are numerous countries where interRAI assessment instruments are used and this may represent an opportunity; interRAI instruments with known performance characteristics could be used to conduct measurements within domains (56). Furthermore, when data are available on a population basis there is value in being able to compare study data. It is estimated that this could be feasible in at least 10 countries at the present (http://www.interrai.org). Further consultation with interRAI and stakeholders of this initiative will be required to determine if this is possible or desirable once the CDEs and COMs domains are finalized.

### Policy considerations

It is important to also consider policy perspectives around this issue. Frailty challenges the engagement of policy-makers because of the large number of instruments/measures and lack of consistency across studies (57). Governmental policy-makers and decision-makers will be key groups to engage. The adoption of a common set of measures will result in better data and an improved understanding of frailty and its impact. Better insights will improve policy formulation, decision making, and how health and social organizations work with frail older adults and their caregivers, thus achieving greater results than simply standardizing data collection for research. Opening a dialogue with policy makers in variety of jurisdictions around the world will be important as this initiative moves forward.

### Better characterization of frail populations

Although a holistic approach to frailty is always going to be required, the group felt that there is a need to better characterize frail populations informed by genetics or biomarkers. An analogy is the evolution in our understanding of diabetes from a type 1/2 classification to a genetically influenced sub-type classification (58). In the future, we may be able to better characterize frailty status based on an individual's biomarker profile (http://www.frailomic.org). As the frailty CDEs and COMs develop over time, frailty biomarkers could be used to additionally characterize frail populations (59, 60). Further research and consultation with the basic science community is required and has been started (61).

### Need to measure burden of the intervention

The burden of an intervention, which likely is different for non-frail older adults versus older adults with frailty, may be an important moderator of clinical effectiveness and cost-effectiveness. Usually non-adherence to the intervention is reported but better measures of burden need to be developed and reported.

## Next Step

An executive committee for this initiative has been convened with international representation. The main objective of this committee is to identify key partners around the world and develop a process for consensus building. The committee will guide the design of the international Delphi process, including the elements of the survey and its broad distribution.

*Conflict of interest:* Dr. Muscedere reports that he is the Scientific Director of the Canadian Frailty Network which is funded by the Government of Canada. Dr. Kim reports that he is the Assistant Scientific Director of the Canadian Frailty Network which is funded by the Government of Canada. Dr. Rockwood reports personal fees from Clinical Cardio Day-Cape Breton University, personal fees from CRUIGM -Montreal, personal fees from Speaker at Jackson Lab, Bar Harbor, MA, personal fees from Speaker at MouseAge, Rome Italy, personal fees from Lundbeck, personal fees from Frontemporal Dementia Study-Group, personal fees from SunLife Insurance, Japan, outside the submitted work; and Kenneth Rockwood is President and Chief Science Officer of DGI Clinical, which in the last five years has contracts with pharma and device manufacturers (Baxter, Baxalta, Shire, Hollister, Nutricia, Roche, Otsuka) on individualized outcome measurement. In 2017 he attended an advisory board meeting with Lundbeck. Otherwise any personal fees are for invited guest lectures and academic symposia, received directly from event organizers, chiefly for presentations on frailty. He is Associate Director of the Canadian Consortium on Neurodegeneration in Aging, which is funded by the Canadian Institutes of Health Research, and with additional funding from the Alzheimer Society of Canada and several other charities, as well as, in its first phase (2013–2018), from Pfizer Canada and Sanofi Canada. He receives career support from the Dalhousie Medical Research Foundation as the Kathryn Allen Weldon Professor of Alzheimer Research, and research support from the Canadian Institutes of Health Research, the QEII Health Science Centre Foundation, the Capital Health Research Fund and the Fountain Family Innovation Fund of the QEII Health Science Centre Foundation. All remaining authors have nothing to disclose.
